# Supplementary material for: Analysis of PPARγ Signaling Activity in Psoriasis
Source: Int J Mol Sci. 2021 Aug 10;22(16):8603. doi: 10.3390/ijms22168603 (PMC8395241; doi:10.3390/ijms22168603)
Supplement: Supplementary file 1 [file ijms-22-08603-s001.zip › Supplemental materials_Analysis of PPARg signaling activity in psoriasis/Pathway models/Models images and html files/Model of PPARG signaling in psoriasis/24019.html]

LPIN1


# Protein LPIN1

|  |  |
| --- | --- |
| URN | urn:agi-llid:23175 |
| Total Entities | 0 |
| Connectivity | 808 |
| Name | LPIN1 |
| Description | lipin 1 |
| Notes | This gene encodes a magnesium-ion-dependent phosphatidic acid phosphohydrolase enzyme that catalyzes the penultimate step in triglyceride synthesis including the dephosphorylation of phosphatidic acid to yield diacylglycerol. Expression of this gene is required for adipocyte differentiation and it also functions as a nuclear transcriptional coactivator with some peroxisome proliferator-activated receptors to modulate expression of other genes involved in lipid metabolism. Mutations in this gene are associated with metabolic syndrome, type 2 diabetes, acute recurrent rhabdomyolysis, and autosomal recessive acute recurrent myoglobinuria (ARARM). This gene is also a candidate for several human lipodystrophy syndromes. [provided by RefSeq, Mar 2017] |

---

|  |  |
| --- | --- |
| Pathway | mTOR Signaling |
|  | MTOR/TP53 Regulates Cell Metabolism |
|  | Lipogenesis Regulation in Adipocyte |
|  | Berardinelli-Seip Syndrome Progression (Hypothesis) |
|  | Proteins Involved in Diabetic Neuropathy |
|  | Proteins Involved in Insulin Resistance |
|  | Proteins Involved in non-Alcoholic Fatty Liver Disease |
|  | Gluconeogenesis Impairment in non-Alcoholic Fatty Liver Disease |
|  | Adipocyte Hypertrophy and Hyperplasia |
|  | Proteins Involved in Polycystic Ovary Syndrome |
|  | Lipodystrophy, Familial Partial |
|  | Lipogenesis/Lipolysis Activation in Cancer Cells and Cancer-Associated Adipocytes |
|  | Insulin -> CEBPA/CTNNB/FOXA/FOXO Expression Targets |
|  | PPAR Psoriasis |
|  | Model of PPARG signaling in psoriasis |
|  | 1\_Triglyceride storage in NAFLD |

---

|  |  |
| --- | --- |
| MedScan ID | 23175 |

---

|  |  |
| --- | --- |
| LocusLink ID | 23175 |
|  | 14245 |
|  | 313977 |

---

|  |  |
| --- | --- |
| Alias | cDNA sequence AF180471 |
|  | phosphatidate phosphatase LPIN1 |
|  | KIAA0188 |
|  | LPIN1 |
|  | 4631420P06 |
|  | mKIAA0188 |
|  | DKFZp781P1796 |
|  | AF180471 |
|  | fld |
|  | lipin 1 |
|  | LPIN I |
|  | OTTHUMP00000179151 |
|  | fatty liver dystrophy |
|  | lipin I |
|  | OTTHUMP00000179153 |
|  | PAP1 |
|  | Lipin1 |
|  | fatty liver dystrophy protein |
|  | lipin-1 |

---

|  |  |
| --- | --- |
| EC Number | 3.1.3.4 |

---

|  |  |
| --- | --- |
| GO ID | 0008195 |
|  | 0003713 |
|  | 0031100 |
|  | 0032869 |
|  | 0016311 |
|  | 0009062 |
|  | 0007077 |
|  | 0006656 |
|  | 0006646 |
|  | 0120162 |
|  | 0045944 |
|  | 0019432 |
|  | 0006642 |
|  | 0005737 |
|  | 0005829 |
|  | 0005789 |
|  | 0005741 |
|  | 0005635 |
|  | 0031965 |
|  | 0005654 |
|  | 0005634 |
|  | 0001085 |
|  | 0042826 |
|  | 0016787 |
|  | 0042975 |
|  | 0031532 |
|  | 0044255 |
|  | 0045444 |
|  | 0006955 |
|  | 0006629 |
|  | 0000266 |
|  | 0000122 |
|  | 0031065 |
|  | 0045598 |
|  | 0006357 |
|  | 0031529 |
|  | 0005783 |
|  | 0016020 |
|  | 0005739 |
|  | 0005667 |
|  | 0006351 |
|  | 0006355 |

---

|  |  |
| --- | --- |
| KEGG ID | mmu:14245 |
|  | hsa:23175 |

---

|  |  |
| --- | --- |
| Organism | Homo sapiens {Organism urn:agi-taxid:9606} |
|  | Mus musculus {Organism urn:agi-taxid:10090} |
|  | Rattus norvegicus {Organism urn:agi-taxid:10116} |
|  | Homo sapiens |
|  | Mus musculus |
|  | Rattus norvegicus |

---

|  |  |
| --- | --- |
| Mouse chromosome position | 12 7.9 cM |

---

|  |  |
| --- | --- |
| OMIM ID | 605518 |
|  | 268200 |

---

|  |  |
| --- | --- |
| Rat chromosome position | 6q16 |
|  | 6q15 |

---

|  |  |
| --- | --- |
| Hugo ID | 13345 |

---

|  |  |
| --- | --- |
| Human chromosome position | 2p25.1 |

---

|  |  |
| --- | --- |
| Swiss-Prot Accession | Q14693 |
|  | Q14693.2 |
|  | A0A1Y7VLN4 |
|  | Q8CD95 |
|  | Q91ZP3 |
|  | E9QKQ5 |
|  | Q91ZP3.1 |
|  | Q5XIM8 |
|  | Q9CQI2 |
|  | Q9JLG6 |
|  | A8MU38 |
|  | B4DET9 |
|  | B4DGS4 |
|  | B4DGZ6 |
|  | B5MC18 |
|  | B7Z858 |
|  | D6W506 |
|  | E7ESE7 |
|  | F5GY24 |
|  | Q53T25 |
|  | A0A0R4J0V3 |

---

|  |  |
| --- | --- |
| GenBank ID | NC\_000002 |
|  | NR\_146080 |
|  | NM\_001261428 |
|  | NP\_001248357 |
|  | NM\_001349208 |
|  | NP\_001336137 |
|  | NM\_001349207 |
|  | NP\_001336136 |
|  | XM\_011510333 |
|  | XP\_011508635 |
|  | XM\_017003623 |
|  | XP\_016859112 |
|  | XM\_024452762 |
|  | XP\_024308530 |
|  | NM\_001261427 |
|  | NP\_001248356 |
|  | XM\_006711872 |
|  | XP\_006711935 |
|  | XM\_011510334 |
|  | XP\_011508636 |
|  | XM\_011510335 |
|  | XP\_011508637 |
|  | XM\_017003627 |
|  | XP\_016859116 |
|  | XM\_017003628 |
|  | XP\_016859117 |
|  | XM\_011510336 |
|  | XP\_011508638 |
|  | XM\_006711870 |
|  | XP\_006711933 |
|  | XM\_024452763 |
|  | XP\_024308531 |
|  | NM\_001349204 |
|  | NP\_001336133 |
|  | NM\_001349202 |
|  | NP\_001336131 |
|  | NM\_001349200 |
|  | NP\_001336129 |
|  | NM\_001349199 |
|  | NP\_001336128 |
|  | NM\_001349206 |
|  | NP\_001336135 |
|  | NM\_001349203 |
|  | NP\_001336132 |
|  | NM\_001349201 |
|  | NP\_001336130 |
|  | NM\_145693 |
|  | NP\_663731 |
|  | XM\_017003624 |
|  | XP\_016859113 |
|  | XM\_017003629 |
|  | XP\_016859118 |
|  | NM\_001349205 |
|  | NP\_001336134 |
|  | XM\_017003625 |
|  | XP\_016859114 |
|  | XM\_017003630 |
|  | XP\_016859119 |
|  | XM\_017003631 |
|  | XP\_016859120 |
|  | NG\_012843 |
|  | AC012456 |
|  | AAY14695 |
|  | AC106875 |
|  | CH471053 |
|  | EAX00918 |
|  | EAX00919 |
|  | EAX00920 |
|  | HI573914 |
|  | CBX47446 |
|  | AF147446 |
|  | AI379040 |
|  | AK127039 |
|  | AK290235 |
|  | BAF82924 |
|  | AK293787 |
|  | BAG57200 |
|  | AK294742 |
|  | BAG57885 |
|  | AK294853 |
|  | BAG57957 |
|  | AK302922 |
|  | BAH13844 |
|  | AK310450 |
|  | AL357194 |
|  | BC018071 |
|  | BC030537 |
|  | AAH30537 |
|  | BI459149 |
|  | BP192775 |
|  | CR749806 |
|  | CAH18666 |
|  | D80010 |
|  | BAA11505 |
|  | DB086946 |
|  | DB153243 |
|  | DC334711 |
|  | Q14693 |
|  | NC\_000078 |
|  | NM\_172950 |
|  | NP\_766538 |
|  | NM\_015763 |
|  | NP\_056578 |
|  | NM\_001130412 |
|  | NP\_001123884 |
|  | XM\_006514975 |
|  | XP\_006515038 |
|  | XM\_006514977 |
|  | XP\_006515040 |
|  | XM\_006514988 |
|  | XP\_006515051 |
|  | XM\_006514978 |
|  | XP\_006515041 |
|  | XM\_006514976 |
|  | XP\_006515039 |
|  | XM\_006514982 |
|  | XP\_006515045 |
|  | XM\_006514987 |
|  | XP\_006515050 |
|  | XM\_006514983 |
|  | XP\_006515046 |
|  | XM\_006514984 |
|  | XP\_006515047 |
|  | XM\_011243815 |
|  | XP\_011242117 |
|  | XM\_006514986 |
|  | XP\_006515049 |
|  | NM\_001355598 |
|  | AC122228 |
|  | AC159631 |
|  | CH466582 |
|  | EDK98473 |
|  | EDK98474 |
|  | AA154452 |
|  | AB093214 |
|  | BAC41398 |
|  | AF180471 |
|  | AAF44296 |
|  | AF412811 |
|  | AAL07798 |
|  | AK009789 |
|  | AK014526 |
|  | BAB29412 |
|  | AK019539 |
|  | BAB31786 |
|  | AK030921 |
|  | BAC27184 |
|  | AK036250 |
|  | AK141489 |
|  | AK154837 |
|  | BAE32865 |
|  | AK180700 |
|  | AK203902 |
|  | BC042462 |
|  | AAH42462 |
|  | BY127780 |
|  | BY209929 |
|  | BY227625 |
|  | CK621866 |
|  | CX205142 |
|  | Q91ZP3 |
|  | NC\_005105 |
|  | XM\_008764593 |
|  | XP\_008762815 |
|  | NM\_001012111 |
|  | NP\_001012111 |
|  | AC\_000074 |
|  | AABR07063682 |
|  | AABR07063683 |
|  | AABR07063684 |
|  | AAHX01042892 |
|  | AAHX01042893 |
|  | AAHX01042894 |
|  | AAHX01042895 |
|  | CH473947 |
|  | EDM03131 |
|  | EDM03132 |
|  | BC083651 |
|  | AAH83651 |
|  | XM\_006711869 |
|  | XP\_006711932 |
|  | XM\_011510337 |
|  | XP\_011508639 |
|  | XM\_011510338 |
|  | XP\_011508640 |
|  | NM\_001261429 |
|  | NP\_001248358 |
|  | XM\_006711871 |
|  | XP\_006711934 |
|  | XM\_006711874 |
|  | XP\_006711937 |
|  | NC\_018913 |
|  | AMYH02003292 |
|  | AMYH02003293 |
|  | DQ894676 |
|  | ABM85602 |
|  | XM\_006514985 |
|  | XP\_006515048 |
|  | XM\_006514980 |
|  | XP\_006515043 |
|  | AC\_000034 |
|  | AAHY01097875 |

---

|  |  |
| --- | --- |
| Swiss-Prot ID | LPIN1\_MOUSE |
|  | LPIN1\_HUMAN |

---

|  |  |
| --- | --- |
| Cell Localization | Mitochondrion outer membrane |
|  | Cytoplasm |
|  | Nucleus membrane |
|  | Nucleus |
|  | Endoplasmic reticulum membrane |
|  | Cytosol |
|  | Cytoplasm, cytosol |

---

|  |  |
| --- | --- |
| Ensembl ID | ENSG00000134324 |
|  | ENSP00000401522.2 |
|  | ENST00000425416.6 |
|  | ENSP00000397908.2 |
|  | ENST00000449576.6 |
|  | ENSP00000256720.2 |
|  | ENST00000256720.6 |
|  | ENSP00000379404.2 |
|  | ENST00000396097.5 |
|  | ENSP00000379405.1 |
|  | ENST00000396098.5 |
|  | ENSMUSG00000020593 |
|  | ENSMUSP00000152276.1 |
|  | ENSMUST00000222989.1 |
|  | ENSMUSP00000070583.5 |
|  | ENSMUST00000067124.5 |
|  | ENSMUSP00000152466.1 |
|  | ENSMUST00000221230.1 |
|  | ENSMUSP00000106696.3 |
|  | ENSMUST00000111067.9 |
|  | ENSRNOG00000004377 |
|  | ENSRNOP00000005863.4 |
|  | ENSRNOT00000005863.6 |
|  | ENSP00000401522 |
|  | ENST00000425416 |
|  | ENSP00000397908 |
|  | ENST00000449576 |
|  | ENSP00000379405 |
|  | ENST00000396098 |
|  | ENSP00000256720 |
|  | ENST00000256720 |
|  | ENSP00000379406 |
|  | ENST00000396099 |
|  | ENSP00000379404 |
|  | ENST00000396097 |
|  | ENSMUSP00000106696 |
|  | ENSMUST00000111067 |
|  | ENSMUSP00000070583 |
|  | ENSMUST00000067124 |
|  | ENSRNOP00000005863 |
|  | ENSRNOT00000005863 |

---

|  |  |
| --- | --- |
| MGI ID | MGI:1891340 |

---

|  |  |
| --- | --- |
| RGD ID | 1307646 |

---

|  |  |
| --- | --- |
| Unigene ID | Hs.467740 |
|  | Hs.739141 |
|  | Mm.153625 |
|  | Rn.229137 |

---

|  |  |
| --- | --- |
| Homologene ID | 9266 |

---
